# Use of programme theory to understand the differential effects of interventions across socio-economic groups in systematic reviews—a systematic methodology review

**DOI:** 10.1186/s13643-017-0638-9

**Published:** 2017-12-29

**Authors:** Michelle Maden, Alex Cunliffe, Naoimh McMahon, Andrew Booth, Gina Michelle Carey, Suzy Paisley, Rumona Dickson, Mark Gabbay

**Affiliations:** 1 Liverpool Reviews and Implementation Group (LRIG), Department of Health Services Research, University of Liverpool, Second Floor, Whelan Building, The Quadrangle, Brownlow Hill, Liverpool, L69 3GB UK; 2 Faculty of Health and Wellbeing, University of Central Lancashire, Brook Building, Preston, PR1 2HE UK; 3 Evidence Based Information Practice, School of Health and Related Research (ScHARR), University of Sheffield, Regent Court, 30 Regent Street, Sheffield, S1 4DA UK; 4 University of Central Lancashire, Preston, Lancashire, PR1 2HE UK; 5 Innovation and Knowledge Transfer (IKT), ScHARR, University of Sheffield, Sheffield, UK; 6 Department of Health Services Research, University of Liverpool, Block B Waterhouse Building, 1–5 Brownlow St., Liverpool, L69 3GL UK

**Keywords:** Systematic review, Equity, Methodology, Theory, Programme theory

## Abstract

**Background:**

Systematic review guidance recommends the use of programme theory to inform considerations of *if* and *how* healthcare interventions may work differently across socio-economic status (SES) groups. This study aimed to address the lack of detail on *how* reviewers operationalise this in practice.

**Methods:**

A methodological systematic review was undertaken to assess if, how and the extent to which systematic reviewers operationalise the guidance on the use of programme theory in considerations of socio-economic inequalities in health. Multiple databases were searched from January 2013 to May 2016. Studies were included if they were systematic reviews assessing the effectiveness of an intervention and included data on SES. Two reviewers independently screened all studies, undertook quality assessment and extracted data. A narrative approach to synthesis was adopted.

**Results:**

A total of 37 systematic reviews were included, 10 of which were explicit in the use of terminology for ‘programme theory’. Twenty-nine studies used programme theory to inform both their a priori assumptions and explain their review findings. Of these, 22 incorporated considerations of both *what* and *how* interventions do/do not work in SES groups to both predict and explain their review findings. Thirteen studies acknowledged 24 unique theoretical references to support their assumptions of what or how interventions may have different effects in SES groups. Most reviewers used supplementary evidence to support their considerations of differential effectiveness. The majority of authors outlined a programme theory in the “Introduction” and “Discussion” sections of the review to inform their assumptions or provide explanations of *what* or *how* interventions may result in differential effects within or across SES groups. About a third of reviews used programme theory to inform the review analysis and/or synthesis. Few authors used programme theory to inform their inclusion criteria, data extraction or quality assessment. Twenty-one studies tested their a priori programme theory.

**Conclusions:**

The use of programme theory to inform considerations of *if*, *what* and *how* interventions lead to differential effects on health in different SES groups in the systematic review process is not yet widely adopted, is used implicitly, is often fragmented and is not implemented in a systematic way.

**Electronic supplementary material:**

The online version of this article (10.1186/s13643-017-0638-9) contains supplementary material, which is available to authorized users.

## Background

A key challenge facing systematic reviewers when complying with recent guidance [1–6] on incorporating considerations of socio-economic health inequalities, is to determine not only *if* but also *how* the interventions being reviewed may work differently across socio-economic status (SES) groups. An understanding of how socio-economic health inequalities may impact on intervention effectiveness can help reviewers to decide whether interventions are likely to have either a positive or negative effect on the health inequality gap [7]. This may then influence their decision on whether or not to include considerations of socio-economic health inequalities in the review.

Guidance on incorporating considerations of health inequalities in systematic reviews recognises the limitations of using traditional approaches to formulate review questions [5]. While the traditional ‘PICO’ (population, intervention, comparison and outcome) framework, and subsequent derivatives [8], can help reviewers to clarify the specific components under review, they are not designed to help to identify explanatory relationships for *if* and *how* interventions may have differential effects on health across different SES groups [9]. For example, when defining the ‘P’ in PICO (i.e. population group), the emphasis is on describing what population characteristics are under review (e.g. condition, age), rather than the way different people experience the healthcare system within which an intervention is delivered.

As a consequence, equity review guidance recommends using additional methods such as programme theory, logic models or theories of change to understand the assumptions behind *if* and *how* the intervention may work differently across SES groups [1, 5]. When considering the need to incorporate health inequalities in systematic reviews, therefore, reviewers need to know *if*, *what* and *how* interventions designed to improve the health of a population may have differential effects across different SES groups. Little is known, however, on if and how reviewers operationalise the equity review guidance when deciding whether or not to incorporate considerations of socio-economic health inequalities in systematic reviews.

### Defining programme theory

Programme theory is the overarching theory or model of how an intervention is expected to work [10]. There is, however, a lack of consistency in the way in which the terms relating to programme theory are applied in the literature, with some authors using them synonymously. Others note that while an overlap between the terms exists, a distinction can nevertheless be made between them [11, 12] (see Table 1).

**Table 1:** Defining programme theory

Programme theory: A programme theory is the overarching theory or model of how an intervention is expected to work. The ‘theory’ in a programme theory “can be an articulation of practice wisdom or of tacit assumptions – that is, not only a formal, research-based theory” ([11], p. 33). A programme theory is made up of two components, a theory of change and a theory of action.	
Theory of change: A theory of change explains the causal processes or hypothesised mechanisms that lead from activities to outcomes [12].	
Theory of action: A theory of action details what the programme or intervention will do in order to activate the change theory [11].	
Logic model: A logic model is a graphical representation of a programme theory, which maps out the links between the intervention and anticipated outcomes.	

The use of programme theory in guiding the conduct of systematic reviews is not new [13–15]. New theory-informed approaches to systematic reviews (e.g. realist reviews, ‘best fit’ framework synthesis) have increased awareness of the use of theory within the systematic review process [16]. More recently, programme theory has been advocated as a tool to help reviewers of complex interventions to better understand ‘what works, for whom and under what circumstance’ [9, 17]. The Cochrane Collaboration have recently published guidance on the choice and use of theory in complex intervention reviews [16].

### Limitations of the systematic review guidance in helping reviews to operationalise a programme theory

While current equity guidance clearly presents the rationale for incorporating considerations of health inequalities in systematic reviews, it offers little practical guidance on how to operationalise a programme theory to inform an understanding of *if*, *what* and *how* interventions work for different SES groups [18].

A study of systematic review guidance for incorporating health inequalities [18] found that of 20 guidance documents, only one [6] looked at how reviewers were operationalising such decisions. Although data were collected on whether reviewers operationalised their decisions by using theory, empirical evidence or personal experience, no information was sought on which theories or evidence was used, or how these were used to inform the review process.

Much of the guidance on incorporating considerations of health inequalities in systematic reviews is written either from the perspective that health inequalities have already been identified as the focus of the review or written with an underlying assumption that reviewers already have a good understanding of health inequalities and how they *could* impact on their review findings [18]. Furthermore, the use of terms, such as ‘programme theory’, ‘logic model’, etc., may neither be widely accepted nor understood by reviewers [1, 19]. These challenges make operationalising the guidance difficult for both expert and novice reviewers who either do not have a health inequality background or are unfamiliar with the use of programme theory to inform systematic reviews.

### Perceived value of programme theory use in systematic reviews

The perceived value of using programme theory to inform systematic reviews is well documented in the literature (see Table 2). From an implementation perspective, programme theory may help to identify the elements of an intervention that may be more effective for given populations, therefore increasing the applicability and usefulness in translating the review findings into practice.

**Table 2:** Perceived value of programme theory to inform systematic reviews [9, 12, 16, 19–21, 76]

• Provide a theoretical basis for the review	
• Aid reviewers in thinking conceptually to gain an initial understanding of the way in which the intervention is likely to work	
• Assist in refining the review question and defining the scope of the review	
• Identify points of uncertainty and provide the rationale for data collection and approach to synthesis	
• Increase the transparency of the review process	

This is particularly important for considerations of health inequalities. Given the diverse nature of health problems and the necessary interactions required between what are often complex interventions and individuals, it is likely that the underlying mechanisms supporting or undermining the effectiveness of interventions will vary and be context dependent [20]. In the event of a deficiency or absence of evidence from review findings, programme theory can help reviewers to make assumptions about whether and how the intervention *may* indirectly result in differential effectiveness, which can then better inform the direction of future health inequalities research [21].

### Operationalising programme theory in systematic reviews

Few empirical papers examine how reviewers utilise programme theory. A recent study by Kneale et al. [12] on the use of programme theory found that only five Cochrane Reviews published between September 2013 and September 2014, and 13 reviews published in the 3ie (International Initiative for Impact Evaluation) database of systematic reviews in 2013, mentioned use of either a logic model or theory of change. All of the reviews included in the Kneale et al. [12] study used programme theory to describe how the intervention might work a priori, but relatively few used it to inform other elements of the review process such as guiding selection criteria or to structure the synthesis. The study authors identified a need to develop good practice on how to use programme theory, logic models and theory of change in systematic reviews to avoid their use becoming merely a ‘tick-box exercise’. The conclusions of their study also support calls made elsewhere for researchers to develop a better understanding of the use and value of theory within the systematic review process [16].

However, the Kneale et al. review [12] offers only limited assistance to reviewers who seek to operationalise the use of programme theory, being based upon a relatively small sample of Cochrane and 3ie reviews and relying upon the included reviews explicitly articulating the terms ‘logic model’ or ‘theory of change’. Programme theory may be invoked either explicitly or implicitly without the use of such terms [10].

### Using programme theory to guide action on health inequalities

One example of how programme theory can be operationalised to guide research on health inequalities is the typology of actions to tackle social inequalities in health [22]. Acknowledging the lack of evidence in primary research on the differential effects of interventions on health across SES groups, Whitehead [22] calls for it to be ‘absolutely imperative’ that a theory-based approach is adopted to guide actions on reducing health inequalities.

In particular, Whitehead ([22], p. 476) calls for the best use of ‘intervention programme theories, to come up with plausible mechanisms for bringing about the desired change’. Using programme theory to understand how interventions may work to bring about an improvement in health across disadvantaged populations, Whitehead [22] suggests four levels of action in tackling the underlying causes of health inequalities (see Table 3).

**Table 3:** Typology of actions to reduce health inequalities with underlying programme theory ([22], pp. 474–475)

Level of action^a^	Underlying cause of heath inequality	Underlying programme theory
1) Strengthening individuals (using person-based strategies to improve the health of the most disadvantaged)	A perceived personal deficit, e.g. lack of knowledge, skills, beliefs, self-esteem	Actions that acknowledge positive strengths (i.e. assets and capabilities disadvantaged individuals possess) and remove barriers to achieving them will allow individuals to act in ways that improve their health
2) Strengthening communities (building social cohesion and mutual support to improve the health of disadvantaged communities)	Greater social exclusion, isolation or powerlessness in disadvantaged communities	Fostering social interactions between members of the same community (horizontal interventions) could influence their local environment leading to healthier neighbourhoods. Improving social interactions across society (vertical interventions) produces a less divided society, builds inclusiveness and increases equitable access to resources for health
3) Improving living and working conditions (improving infrastructure and access to services)	Greater exposure to health-damaging living and working environments with declining social position and poorer access to essential goods and services	Improving the physical environment and addressing psychosocial health hazards have the potential to improve the health of the whole population especially that of people living in the poorest conditions, thereby reducing the gradient in health
4) Promoting healthy macro policies (making structural alterations to economic, cultural and environmental conditions to influence the standard of living of the whole population)	The standard of living, income, unemployment, job security, etc., are linked to wider macro-economic, cultural and environment conditions	Universal actions that aim to alter the macro-environment or cultural environment to reduce poverty span several sectors and work across the whole population. These actions are potentially more efficient in reducing poverty and tackling the socio-economic gradient

^a^The levels of action are based on the widely cited Dahlgren and Whitehead [77] conceptual model of the social determinants of health

Even though health inequalities research includes examples of the use of programme theory, there has been no exploration, to date, of its use in informing considerations of socio-economic inequalities in health in systematic reviews.

### Aim

The purpose of this study is to assess *if, how* and the *extent* to which systematic reviewers operationalise the guidance on the use of programme theory in considerations of socio-economic inequalities in health.

The objectives are as follows:

i) To identify the extent to which reviewers operationalise the equity guidance in articulating considerations of whether and how differences in intervention effectiveness on health may be expected across SES groups
ii) To identify how reviewers rationalise an understanding of what and how interventions have differential effects in or across SES populations (e.g. use of programme theory terminology and tools, authority for their decision based on theory, empirical evidence, personal experience)
iii) To identify the extent to which reviewers are using programme theory to inform the review process (e.g. to predict or explain a change in health status, to inform the approach to the methods).

## Methods

A systematic methodology review was undertaken. A methodology review is defined by the Cochrane Methodology Reviews Group [23] as ‘examining the evidence on methodological aspects of systematic reviews, randomised trials and other evaluations of health and social care’. The PRISMA guidance for the conduct and reporting of systematic reviews was adhered to during the review process [24].

### Inclusion criteria

Table 4 outlines the inclusion and exclusion criteria.

**Table 4:** Inclusion and exclusion criteria

**Inclusion criteria**	**Further explanation**
Published systematic reviews	With or without meta-analysis
Assessed the effects of a non-pharmacological intervention on health behaviour or health outcome as primary outcome	Health behaviour is defined broadly as ‘any behaviour that may affect an individual’s physical health or any behaviour that an individual believes may affect their physical health’ ([78], p. 94)
Measured or collected data on the effects of SES on the intervention	SES is defined as incorporating a measure of one or more of the following: income, education or occupation
Reported either differential effects relating to SES (universal) or targeted low SES populations (targeted)	
Published between January 2013 and May 2016	The date period is selected to acknowledge the publication in 2012 of the Reporting Guidelines for Systematic Reviews with a Focus on Health Equity [1]
**Exclusion criteria**	**Further explanation**
Included a primary outcome relating to a context other than health or health behaviour	
Did not separate SES data from other equity considerations	For example, if it was not possible to separate data on ethnicity, age, or SES
Did not examine the effectiveness of an intervention	
Measured the effectiveness of pharmacological interventions	
Protocols or primary study designs	
Published in a language other than English	
Full text was not available at the time of data collection and analysis	

*SES* socio-economic status

### Search strategy

A systematic methodology review requires a different approach to the identification of the literature compared with conventional systematic reviews of empirical research [25]. Methods for undertaking methodological reviews are undefined, but the focus of the search should aim for a systematic rather than exhaustive approach [26]. Databases and websites searched were selected for their potential relevance in indexing records that were relevant to the review aims and objectives (see Table 5).

**Table 5:** Databases and websites searched

• MEDLINE (Ovid)	
• CINAHL (EbscoHost)	
• The Cochrane Database of Systematic Reviews (http://www.cochranelibrary.com/) • Centre for Reviews and Dissemination Database (http://www.cochranelibrary.com/)	
• Health Technology Assessments (http://www.cochranelibrary.com/)	
• Database of promoting health effectiveness reviews (DoPHER) (https://eppi.ioe.ac.uk/webdatabases4/Intro.aspx?ID=9)	
• NIHR Journals Library (https://www.journalslibrary.nihr.ac.uk/)	
• Campbell Collaboration Library of Systematic Reviews (https://www.campbellcollaboration.org/library.html)	
• 3ie (International Initiative for Impact Evaluation) database of systematic reviews (http://www.3ieimpact.org/en/evidence/systematic-reviews/)	
• Google Scholar (https://scholar.google.co.uk/)	

The search strategy was developed by one author (MM) with expertise in information retrieval in Ovid MEDLINE and adapted for other electronic databases. A second information scientist reviewed the search strategy. Full-text searches were undertaken in Google Scholar (Additional file 1).

### Data collection and analysis

#### Study selection

A two-stage process to filter studies was undertaken. Stage one involved an initial screening of titles and abstracts against the inclusion criteria. Studies were then categorised into (1) ‘probable’ studies that appeared to meet the inclusion criteria; (2) ‘possible’ studies that may be eligible but further information was required; and (3) excluded studies. Studies in the first two categories were taken forward to stage two where the full text of the study was retrieved and assessed for eligibility. Any studies not meeting the inclusion criteria after stage two were excluded with reasons noted. At least two reviewers (MM, NM, GC) independently screened all studies. Disagreements were resolved by discussion. Covidence.org was used to screen the studies. Multiple publications were analysed as one study.

#### Quality assessment

The approach to quality assessment was guided by the aims of the review and follows the advice of Snilstveit ([27], p. 400) that ‘authors should systematically assess the quality of all studies included in their review, adopting criteria that are sensible for the question it is being used to answer’. No formal criteria exist to assess the use of programme theory in systematic reviews; therefore, all included studies were assessed against PRISMA Equity Extension criteria [1, 5] for reporting use of programme theory (see Table 6).

**Table 6:** Quality assessment criteria based on the PRISMA Equity Extension checklist reporting on programme theory [1, 5]

1. PRISMA rationale (item 3): Describe assumptions about mechanism(s) by which the intervention is assumed to have an impact on health equity. The review should describe a priori how and why interventions are expected to work and the influence of factors such as setting and participant and programme characteristics	
2. Rationale (item 3A): Provide the logic model/analytical framework, if done, to show the pathways through which the intervention is assumed to affect health equity and how it was developed	
3. Discussion/conclusions (item 26): Present extent and limits of applicability (what does/does not work) to disadvantaged populations of interest and describe the evidence and logic (how/why) underlying those judgements	

#### Data extraction and synthesis

Two review authors (MM, AC) extracted data independently from the included studies using pre-determined criteria. The data extraction form was piloted. Disagreements were resolved by discussion. Study characteristics extracted included author, year of publication, review topic, type of synthesis, types of studies included in the reviews, whether the study had an SES focus (where the primary aim related to effects within or across SES groups) or SES was accounted for (e.g. SES data were collected, subgroup analysis was undertaken on SES characteristics), intervention type, population, outcomes (relevant to SES), programme theory terminology used in the review, and use of programme theory to inform the review process. A template, adapted from that of Kneale et al. [12], was used to extract data on the use of programme theory. As this study is an exploration of the use of programme theory, a narrative approach to synthesis was undertaken.

## Results

### Search results

**Fig. 1:**
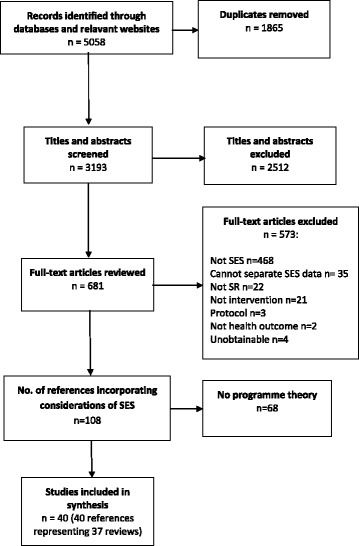
Flowchart of search results

A total of 5058 references were identified from the literature search. One hundred and eight references incorporated considerations of SES, either by collecting data relating to SES variables or by undertaking data analysis on SES variables. Forty references [20, 28–66] reporting on 37 studies (40%) articulated considerations of *if*, *what* or *how* interventions designed to improve the health of a population may have differential effects across different SES groups in systematic reviews and were included in this study (Fig. 1)*.*

### Included study characteristics

Table 7 highlights the characteristics of the included studies. The most reviewed topics were obesity- and diet-related issues. Twenty-eight reviews had an SES focus, whereby the aim or an objective of the review related to assessing either differential effectiveness of interventions across SES populations, or the effectiveness of interventions within a targeted group of socio-economically disadvantaged populations. Nine reviews accounted for effectiveness targeted at, or across, socio-economically disadvantaged populations (e.g. using subgroup analysis) but did not report it as being a specific aim or objective of the review.

**Table 7:** Included study characteristics

Author	Intervention	Population/setting	Outcomes (relevant to SES)	Type of synthesis	Type of studies included	No. of studies included in review	Did review aim to consider ‘what works, for SES’? (Method of analysis)
SES focus							
Backholer et al. [28]	Sugar-sweetened beverage tax	High-income countries	Differential effects on beverage purchases and consumption, weight, amount paid in SSB taxes	Narrative	Any study design	11	–
Bambra et al. [29]^a^; Hillier-Brown et al. [31]	Individual-, community- and societal-level interventions aimed at reducing inequalities in obesity	Children (0–18 years) in any setting in any country	Targeted/differential effects on proxy for body fat (weight and height, BMI, waist measurement/waist-to-hip proportion, percentage body fat content, skinfold thickness, ponderal index in relation to childhood obesity)	Meta-analysis/narrative	RCT, nRCT, prospective/retrospective cohort studies, prospective repeat cross-sectional studies	76 (25 measured differential effects by SES)	✓ (descriptive analysis, sensitivity analysis)
Bambra et al. [29]^a^; Cairns et al. [32]; Hillier-Brown et al. [30]	Individual-, community- and societal-level interventions aimed at reducing inequalities in obesity	Adults (≥ 18 years) in any setting in any country	Targeted/differential effects on proxy for body fat (weight and height, BMI, waist measurement/ waist-to-hip proportion, percentage body fat content, skinfold thickness, ponderal index in relation to childhood obesity)	Meta-analysis/narrative	RCT, nRCT, prospective/retrospective cohort studies, prospective repeat cross-sectional studies	103 (36 measured differential effects by SES)	✓ (descriptive analysis, sensitivity analysis)
Beauchamp et al. [33]	Public health obesity prevention	Interventions addressed everyone across the social gradient	Differential effects on change in anthropometric outcomes	Narrative	Any study design	14	✓ (descriptive analysis)
Boelsen-Robinson et al. [34]	Whole of community obesity prevention	Interventions across different socio- economic strata	Differential effects on behavioural change, energy balance, anthropometric outcomes	Narrative	Any study design	13	✓ (descriptive analysis)
Brown et al. [36]^b, c^; Brown et al. [35]	Community pharmacy delivered interventions focused on alcohol misuse, smoking cessation and weight management	People of any age in any country	Targeted/differential effects on behavioural outcome (e.g. quit rate, change in alcohol intake), weight loss interventions had to report anthropometric outcome	Meta-analysis/narrative	RCTs, nRCTs, CBAs, ITS and repeated measure studies	24	✓ (descriptive analysis)
Brown et al. [37]	Population-level tobacco control	Adults (≥ 18 years) or studies which measured children’s reports of parental smoking in a country at stage 4 of the tobacco epidemic or in the WHO European Region	Differential effects on smoking related outcomes: Social norms/attitudes, exposure to second-hand smoke, policy reach, use of quitting services, quit attempts, smoking prevalence, morbidity	Narrative	All primary study designs, including: RCT, non-RCT, cohort studies, cross-sectional, qualitative	117	–
Brown et al. [38]	Individual-level smoking cessation interventions undertaken in Europe since 1995	Adults (≥ 18 years) based in a WHO European Region country	Differential effects on smoking cessation	Narrative	All primary research designs, including RCT, non-RCT, cohort studies, cross-sectional, qualitative	29	–
Brown et al. [39]	Population-level interventions/policy- and individual-level cessation support	Participants (birth–25 years) in a country in the WHO European Region or non-European country at stage 4 of the tobacco epidemic	Differential effects on smoking-related outcomes: Intentions/ attitudes/perceptions, exposure to second-hand smoke, smoking behaviour, sensitivity to price, initiation, relapse, cessation rates, smoking prevalence, morbidity	Narrative	All primary research designs, including RCT, non-RCT, cohort studies, cross-sectional, qualitative	38	–
Bull et al. [40]	Interventions targeting a change in smoking, eating and/or physical activity behaviours	Adults (≥ 18 years) of low income and from the general population	Behavioural outcomes relevant to smoking cessation, healthy eating and physical activity	Meta-analysis	RCTs and cluster RCTs	35	–
Cleland et al. [41]	Any intervention focused on increasing physical activity	Community-dwelling socio-economically disadvantaged women (19–64 years)	Physical activity outcome, or closely related (e.g. cardiorespiratory fitness)	Meta-analysis	RCTs, nRCTs	19	✓ (subgroup-analysis, meta-regression)
Everson-Hock et al. [42]	Community-based physical activity and dietary	Adults (18–74 years) from a low SES group within the UK	Effectiveness, acceptability	Narrative (mixed-methods)	Quantitative intervention studies, qualitative evaluations of interventions, qualitative studies assessing beliefs and perceptions of physical activity	35	–
Gardner, et al. [43]	Interventions that aimed to increase mammography use	Asymptomatic low-income women	Uptake of mammography	Meta-analysis	RCT	21	✓ (subgroup and meta-regression)
Hill et al. [44]	Tobacco control	Adults (≥ 18 years) in countries at an advanced stage of the tobacco epidemic	Targeted/differential effects on smoking related outcomes	Narrative	Reviews and primary research	84	–
Hollands et al. [45]^c^	Portion, package or tableware size	Adults and children directly engaged with manipulated products	Differential effects on behavioural outcomes (consumption or selection of food, alcohol, or tobacco products)	Meta-analysis/narrative	RCTs	70	✓ (meta-regression)
Kader et al. [46]	Universal parental support targeting children’s health behaviours	At least one parent/caregiver of a child 2–18 years. with or without their child	Targeted/differential effects on children’s dietary habits, physical activity, sedentary behaviour, weight status	Narrative	Prospective studies assessing effectiveness of a controlled intervention	35 (6 with SES focus)	–
Kendrick et al. [47]	Home safety interventions	Children and young people (≤ 19 years) and their families	Differential effects on self-reported or medically attended injury in children/young people	Meta-analysis (IPD)/narrative	RCTs, nRCTs, CBA	98	–
Kristjansson et al. [48]	Supplementary feeding	Children (3 months–5 years) from socio-economically disadvantaged groups or all socio-economic groups with results stratified by SES	Targeted/differential effects on physical (growth), psychosocial health in children	Meta-analysis/narrative	RCTs, c-RCTs, CCT, CBA, ITS	32	✓ (sub-group analysis and process evaluation)
Laba et al. [49]	Strategies to increase patient adherence to cardiovascular medications	Socioeconomically disadvantaged adults with prescribed medications for prevention/treatment of cardiovascular disease	Targeted/differential effects on patient adherence	Narrative	RCTs, quasi-RCTs	14	✓ (descriptive analysis)
Laws et al. [50]	Obesity prevention	Healthy children (0–5 years from) socioeconomically disadvantaged or Indigenous families	Targeted/differential effects on anthropometric measures, child/family diet, parental feeding practices related to obesity, physical activity, sedentary behaviours	Narrative	Any study design	32	✓ (descriptive analysis)
Magnee et al. [51]	Obesity prevention	Participants included within studies identified from a systematic inventory (1990–2007) of Dutch obesity prevention interventions	Differential effects on anthropometric measures, obesity-related behavioural outcomes (e.g. diet, physical activity)	Narrative	Studies selected from a systematic inventory (1990–2007) of Dutch obesity prevention interventions	26	–
McGill et al. [52]	Promotion of healthy eating	Healthy populations (any age/gender)	Differential effects on dietary intake	Narrative	Any study design measuring effects of intervention	36	✓ (descriptive analysis)
McLean et al. [20]^d^	Reminder systems for scheduled health service encounters	Examined differential effectiveness across particular subgroups of the population (age, gender, ethnic group, SES, etc.)	Differential effects on improving uptake	Thematic/narrative	Effectiveness review: RCTs, SRs Realist informed review: studies examining effectiveness of outpatient appointment reminders, qualitative/quantitative designs on appointment attendance behaviour, studies of adherence to treatment, theories/ models/frameworks relating to appointment attendance	Effectiveness review: 42 Realist informed review: 463	✓ (descriptive analysis, realist informed review)
Mizdrak et al. [53]	Food/beverage price change	NR	Differential response in purchase of targeted foods	Narrative	Controlled experimental study	8	✓ (descriptive analysis)
Moore et al. [54]	Universal school-based health behaviour	School children (4–18 years)	Differential effects on diet, physical activity, smoking, alcohol	Narrative	RCTs, quasi-experimental studies	20	✓ (content analysis)
Moredich et al. [55]	Physical activity and weight loss	Low-income adult women	Change in weight	Integrative	Intervention studies	7	✓ (descriptive analysis)
Rojas-Garcia et al. [56]	Healthcare interventions to treat depressive disorders	Socially disadvantaged patients with depressive disorders	Reduction of depressive symptoms	Meta-analysis/narrative	Controlled trials including RCTs & quasi-experimental studies	15	✓ (meta-regression)
Sarink et al. [57]	Menu labelling	Adolescents or adults of a low SEP population or analysis stratified by a measure of SEP	Targeted/differential effects on awareness of exposure, understanding, food or energy purchased or consumed, body mass index	Narrative	Quantitative and qualitative	18	–
SES accounted for							
Ciciriello et al. [58]^e^	Multimedia-based patient education about prescribed or over the counter medications	People of all ages prescribed a particular medication or medication regimen or who had obtained an over-the-counter medication	Patient or carer knowledge about the medication, any measure of skill acquisition related to the medication	Meta-analysis/narrative	RCTs, quasi-RCTs	24	–
Ejemot-Nwadiaro et al. [59]^e^	Hand-washing promotion	Adults and children in day care centres or schools, patients in hospitals, communities or households	Episodes of diarrhoea	Meta-analysis/narrative	RCTs, cluster RCTs	22	–
Gittelsohn et al. [60]	Community-based prepared food sources	NR	Access to and consumption of healthful foods (psychosocial factors (awareness, knowledge, acceptability), behaviour, frequency of use, frequency of purchase, increase in healthful food sales)	Narrative	Some form of written documentation that included a description of the intervention and evaluation	19	–
Gurol-Urganci et al. [61]^e^	Mobile phone messaging reminders	All study participants, regardless of age, gender, ethnicity	Rate of attendance at healthcare appointments	Meta-analysis	RCTs	8	–
Hartmann-Boyce et al. [62]	Self-help	Adults (≥ 18 years) with body mass index ≥ 25 kg/m^2^	Targeted/differential effects on change in weight	Meta-analysis/narrative	RCTs	23	✓ (meta-regression)
Kroon et al. [63]	Structured self-management education programmes for osteoarthritis (OA)	People diagnosed with OA	Self-management of OA, participant’s positive and active engagement in life, pain, global OA scores, self-reported function, quality of life, withdrawals	Meta-analysis	RCTs, quasi-RCTs	29	–
Lutge et al. [64]^c^	Any material inducement to return for TB test results or adhere to or complete anti-TB preventive or curative treatment	People receiving curative treatment for active TB, people receiving preventive therapy for latent TB or people suspected of TB undergoing, and collecting results of, diagnostic tests	Cure or completion of treatment, cases of active TB; completion of prophylactic treatment, number returning to collect test results within the appropriate time frame for that test.	Meta-analysis/narrative	RCTs	12	–
Pega et al. [65]^c^	In-work tax credits	Working age adults (18–64 years)	Self-rated general health, mental health or physical distress, mental illness, overweight and obesity, alcohol use, tobacco use	Narrative	CBA, ITS	5	–
Polec et al. [66]^c^	Interventions that aimed to increase the ownership and appropriate use of insecticide-treated bednets (ITN)	Children and adults with permanent residence in malarial areas	ITN ownership, appropriate ITN use	Meta-analysis/narrative	RCTs, cluster RCTs, non-RCTs, CBA, ITS	10	–

Abbreviations: *SEP* socio-economic position, *SES* socioeconomic status, *RCT* randomised controlled trial, *nRCT* non-randomised controlled trial, *quasi-RCT* quasi-randomised controlled trial, *cluster RCT* cluster randomised controlled trial, *SR* systematic review, *CBA* controlled before-and-after study, *ITS* interrupted time series, *NR* not reported

^a^Two studies reported across four publications

^b^One study reported in two publications

^c^Subgroup analysis planned by SES but not undertaken

^d^One study undertook two systematic reviews relating to effectiveness and ‘what works, for whom, under what circumstance in relation to SES populations

^e^Data collected on SES variables, but no analysis undertaken

Moving beyond simply aiming to measure the effectiveness of an intervention, 18 studies specifically aimed to examine *which* characteristics relating to the intervention may have different effects in and across SES populations. The majority of reviews undertook a narrative synthesis (*n* = 18) with the number of studies included in the reviews ranging from 5 to 463.

Despite articulating a programme theory on how they expected the intervention to work differently for SES populations, three reviews [58, 59, 61] reported only that data were collected on SES characteristics and did not offer any analysis of data by SES. Five reviews [36, 45, 64–66] reported either a lack of data on differential effects by SES within the included studies in the review or a lack of availability of studies for inclusion in the review.

### Quality assessment

Table 8 presents the results of the quality assessment. Only six reviews met all three quality criteria. One review [37] reported using a logic model but did not include it in the review. Of the eight reviews that reported adhering to the PRISMA Equity Extension guidance [1], only one [57] met all three criteria.

**Table 8:** Quality assessment

Author	PRISMA rationale (item 3): Describe assumptions about mechanism(s) by which the intervention is assumed to have an impact on health equity. The review should describe a priori how and why interventions are expected to work and the influence of factors such as setting and participant and programme characteristics	PRISMA rationale (item 3A): Provide the logic model/analytical framework, if done, to show the pathways through which the intervention is assumed to affect health equity and how it was developed	Discussion/conclusions (item 26): Present extent and limits of applicability (what does/does not work) to disadvantaged populations of interest, and describe the evidence and logic (how/why) underlying those judgements
Backholer et al., [28]^a^	✓		✓
Bambra et al., [29]^b, d^	✓	✓	✓
Bambra et al., [29]^c, d^	✓	✓	✓
Beauchamp et al., [35]	✓		✓
Boelsen-Robinson et al., [34]^a^	✓		✓
Brown et al., [36]^d, e^	✓		✓
Brown et al., [37]^a, d^			✓
Brown et al., [38]^a^			✓
Brown et al., [39]^a^			✓
Bull et al., [40]	✓		✓
Ciciriello et al., [58]	✓		✓
Cleland et al., [41]			✓
Ejemot-Nwadiaro et al., [59]	✓		
Everson-Hock et al., [42]	✓		✓
Gardner et al., [43]			✓
Gittelsohn et al., [60]	✓		✓
Gurol-Urganci et al., [61]	✓		
Hartmann-Boyce et al., [62]	✓		✓
Hill et al., [44]			✓
Hollands et al., [45]^a, d^	✓	✓	
Kader et al., [46]			✓
Kendrick et al., [47]	✓		
Kristjansson et al., [48]	✓	✓	✓
Kroon et al., [63]			✓
Laba et al., [49]^d^	✓		✓
Laws et al., [50]	✓		✓
Lutge et al., [64]	✓		✓
Magnee et al., [51]	✓		✓
McGill et al., [52]^a^	✓		✓
McLean et al., [20]^d^	✓	✓	✓
Mizdrak et al., [53]	✓		
Moore et al., [54]	✓		✓
Moredich et al., [55]			✓
Pega et al., [65]^d^	✓	✓	
Polec et al., [66]	✓	✓	✓
Rojas-Garcia et al., [56]	✓		✓
Sarink et al., [57]^a^	✓	✓	✓
Total	28	8	31

^a^Study reports use of PRISMA Equity Extension [1]

^b^Study 1 review in child population [32, 34]

^c^Study 2 review in adult population [32, 33, 35]

^d^Refer to conceptual/casual modelling/behavioural frameworks rather than analytical framework/logic models

^e^Two studies report on same review [38, 39]

### How reviewers rationalise an understanding of if, what and how/why interventions have differential effects in or across socio-economic populations

#### Defining programme theory terminology

Ten studies were explicit in the use of terminology for ‘programme theory’; however, not a single review mentioned the term ‘programme theory’. Two reviews [37, 66] referred to a ‘logic model’, three describe a conceptual model [45, 48, 65], while others referred to a logic pathway [57], conceptual framework [20], casual modelling framework [36] or, simply, framework ([29], child and adult reviews). The remaining studies were implicit, rather than explicit in their use of programme theory, describing their assumptions about *what* and *how* interventions may work differently for different SES populations without labelling it as ‘programme theory’.

#### ‘If’ interventions work/do not work for different socio-economic groups

With the exception of three studies [53, 58, 63], all studies considered whether it was likely that interventions may have differential effects in health within or across SES populations a priori in the “Introduction” section. Such a verdict was largely made on the basis of the burden of disease in different SES groups. For example, the risk or prevalence of the disease was greater for lower SES populations compared with higher SES populations [33]. Others described the burden in terms of higher rates of unhealthy behaviours, such as cigarette consumption [39], or lower rates of healthy behaviours, such as adherence to medications, among lower SES groups [49].

#### ‘What’ interventions work/do not work for different socio-economic groups

All included studies considered *what* types of interventions are likely to work or not work for different SES groups. For example, Bambra et al. [29] suggest that tailored weight-loss interventions worked equally well or better in children in low SES groups. In a review of interventions to improve medication adherence, Laba et al. [49] found that physician- and patient-targeted interventions were most effective in socioeconomically disadvantaged populations. This was in contrast to a previous systematic review [67] which found larger improvements in medication adherence among the general population in physician only-targeted interventions.

Three of these studies [45, 59, 61] incorporated these considerations to inform only their a priori assumptions of what works for different SES groups, and five studies [37, 39, 46, 55, 63] incorporated these considerations only to explain what interventions work based on their review findings. The remaining 29 studies considered what types of interventions work or do not work both in their a priori assumptions and in explaining their review findings. Studies used programme theory to inform considerations of *what works*, as well as *what does not work*.

#### ‘How’ interventions work/do not work for different socio-economic groups

All included studies also considered *how* interventions may or may not work for different SES groups. Of these, six reviews [45, 47, 53, 59, 61, 65] incorporated such considerations only a priori, and nine [37–39, 41, 43, 44, 46, 55, 63] discussed *how* interventions may have differential effects only to explain their review findings.

Twenty-two studies considered *how* interventions work or do not work both in their a priori assumptions and in their explanations of the review findings. For example, Laba et al. [49] suggest that differences in adherence behaviour between social groups can help explain why interventions which target both patient and physician are more effective for lower SES groups. Whereas in a systematic review of obesity-related lifestyle interventions, Magnee et al. [51] suggest that greater effects may be seen in higher SES groups because lower SES groups participate less. They go on to say that lower participation rates in lower SES groups may be due to either intervention design (e.g. recruitment strategies not reaching lower SES groups) or participant response (e.g. lower SES groups may not prioritise participating in interventions if they are experiencing other material and psychosocial problems).Overall, 22 studies incorporated considerations of both *what* and *how* interventions work or do not work in and across SES groups to both predict and explain their review findings.

### Legitimisation of programme theory in systematic reviews

Thirteen studies (see Table 9) referenced the theoretical literature to inform an understanding of what or how/why interventions may lead to differential effectiveness within or across SES groups. One study mentioned the Oxford Food and Activity Behaviours taxonomy but did not provide a reference [62]. The theoretical literature was used to inform both a priori assumptions (*n* = 10) ([20, 29] (child and adult reviews), [33, 36, 40, 49, 52, 54, 62]) and explanations of review findings (*n* = 7) [33, 40, 44, 51, 52, 54, 55]. In four studies [33, 40, 52, 54], the theoretical literature informed both a priori assumptions and explanations of review findings.

**Table 9:** Referenced theoretical literature in systematic reviews exploring the effectiveness of interventions in SES populations

No. of studies	Theoretical literature
5 [29]^a^, [40, 51, 52]	White M, Adams J, Heywood P. How and why do interventions that increase health overall widen inequalities within populations? In: Barbones S, editor. Health, inequality and public health. Volume 65. Bristol: Policy Press; 2009.
5 [29]^a^, [33, 52, 54]	Whitehead M. A typology of actions to tackle social inequalities in health. J Epidemiol Community Health. 2007; 61:473–8. http://dx.doi.org/10.1136/jech.2005.037242
4 [29]^a^, [33, 54]	McLaren L, McIntyre L, Kirkpatrick S. Rose’s population strategy of prevention need not increase social inequalities in health. Int J Epidemiol. 2010; 39:372–7.
2 [29]^a^	Graham H, Kelly M. Health inequalities: concepts, frameworks and policy. London: Health Development Agency; 2004.
2 [29]^a^	European strategies for tackling social inequities in health: levelling up part 2. Available at: http://www.who.int/social_determinants/resources/leveling_up_part2.pdf
2 [29]^a^	Whitehead M, Dahlgren G. Concepts and principles for tackling social inequities in health: levelling up Part 1. Copenhagen: WHO Regional Office for Europe; 2006.
1 [44]	Graham H. Unequal lives: health and socio-economic inequalities. Maidenhead: McGraw-Hill Open University Press; 2007.
1 [62]	Mackenbach JP. The persistence of health inequalities in modern welfare states: the explanation of a paradox. Soc Sci Med. 2012; 75(4):761–9.
1 [64]	Phelan JC, Link BG, Tehranifar P. Social conditions as fundamental causes of health inequalities theory, evidence, and policy implications. J Health Soc Behav. 2010; 51(1 suppl): S28–40.
1 [52]	Frieden TR. A framework for public health action: the health impact pyramid. Am J Public Health. 2010; 100:590–5.
1 [55]	Bandura A. Self-efficacy: toward a unifying theory of behavioural change. Psychol Rev. 1977; 84:191–225.
1 [49]	Michie S, van Stralen MM, West R. The behaviour change wheel: a new method for characterising and designing behaviour change interventions. Implement Sci. 2011; 6:42.
1 [40]	Michie S, Richardson M, Johnston M, Abraham C, Francis J, Hardeman W, et al. The behaviour change technique taxonomy (v1) of 93 hierarchically clustered techniques: building an international consensus for the reporting of behavior change interventions. Ann Behav Med. 2013; 46:81–95.
1 [52]	Grier S, Bryant CA. Social marketing in public health. Annu Rev. Public Health. 2005;26:319–39
1 [36]	Hardeman W, Sutton S, Griffin S, Johnston M, White A, Wareham NJ, et al. A causal modelling approach to the development of theory-based behaviour change programmes for trial evaluation. Health Educ Res. 2005; 20:676–87. http://dx.doi.org/10.1093/her/cyh022
1 [20]	Coomes CM, Lewis MA, Uhrig JD, Furberg RD, Harris JL, Bann CM. Beyond reminders: a conceptual framework for using short message service to promote prevention and improve healthcare quality and clinical outcomes for people living with HIV. AIDS Care. 2012; 24:348–57.
1 [20]	Ajzen I. From intentions to actions: a theory of planned behavior. In: Kuhl J, Beckmann J, editors. Action-control: from cognition to behavior. Heidelberg: Springer; 1985. pp. 11–39.
1 [20]	Prochaska JO, Norcross JC, DiClemente CC. Changing for good: the revolutionary program that explains the six stages of change and teaches you how to free yourself from bad habits. New York, NY: W. Morrow; 1994.
1 [20]	Deci EL, Ryan RM. An overview of self-determination theory. In: Ryan RM, editor. The Oxford handbook of human motivation. Oxford: Oxford University Press; 2012. pp. 85–107.
1 [20]	Phillips KA, Morrison KR, Andersen R, Aday LA. Understanding the context of healthcare utilization: assessing environmental and provider-related variables in the behavioral model of utilization. Health Serv Res. 1998; 33:571–96.
1 [20]	Rogers RW. A protection motivation theory of fear appeals and attitude change. J Psychol. 1975; 91:93–4. http://dx.doi.org/10.1080/00223980.1975.9915803
1 [20]	Glasser W. Choice theory: a new psychology of personal freedom. London: Harper Collins; 2009.
1 [20]	Cooper HC, Geyer R. What can complexity do for diabetes management? Linking theory to practice. J Eval Clin Pract. 2009; 15:761–5. http://dx.doi.org/10.1111/j.1365-2753.2009.01229.x
1 [36]	Nuffield Intervention Ladder. In: Policy process and practice. Public Health: Ethical Issues. London: Nuffield Council on Bioethics; 2009.

*SES* socio-economic status

^a^Two studies reported across four publications [32–35]

Collectively, the included studies acknowledged 24 unique theoretical references to support a priori assumptions and explanations of the review findings of what or how/why interventions may have different effects within and across SES groups (see Table 9). The most referenced were intervention theories: ‘How and why do interventions that increase health overall widen inequalities within populations?’ [68] and ‘A typology of actions to tackle social inequalities in health’ [22].

The majority of the included studies (*n* = 36) used supplementary evidence to support their considerations of differential effectiveness. Supplementary evidence included empirical (qualitative or quantitative), descriptive, or policy-related evidence. Of note here is the use of supplementary evidence to examine intervention-generated inequalities (e.g. [69, 70]). In 32 of the included studies, the authors’ review findings were used to inform explanations of the review findings of differential effectiveness. Only two studies [20, 66] mentioned the involvement of other stakeholders in developing their logic models or programme theory. In some studies, only partial support for the programme theory was derived either from the literature or from the review findings.

### Extent of use of programme theory to inform the review process

Table 10 outlines the extent to which programme theory is used to inform the review process within the included studies. The majority of authors outlined a programme theory in the “Introduction” and “Discussion” sections of the review to inform their assumptions (*n* = 32) or to provide explanations (*n* = 34) of what or how interventions may result in differential effects within or across SES groups. Despite not always being explicit in their use of programme theory, 29 review teams used this approach to inform both their a priori assumptions and explanations of review findings.

**Table 10:** Use of programme theory in systematic reviews of effectiveness of interventions in SES populations

	Review initiation: Indicate whether reported that PT is used to communicate aims of review in engaging with stakeholder or involving/recruiting different team members or obtaining funding	Review question/methodology: Indicate whether reported that PT is based on, or adapted from, existing tools/theories	Review question/background: Indicate assumptions on what intervention(s) may be likely to work/not work for SES populations? (a priori PT)	Review question/background: Indicate assumptions on how/why intervention(s) may be likely to work/not work for SES populations? (a priori PT)	Search strategy (selection criteria): Indicate whether reported that PT is used to make decisions on the inclusion criteria for studies in the review	Description of study characteristics: Indicate whether reported that PT is used to make decisions on coding information on study characteristics (data extraction)	Quality and relevance assessment: Indicate whether reported that PT is used as reference point in choosing quality assessment tools	Used to guide analyses: State that they specifically used their PT of how the intervention may work to guide the analysis	Synthesis: Present their synthesis based on their PT	Discussion/Conclusion: Use programme theory to explain what intervention(s) may be likely to work/not work for SES populations at the end of the review to explain their findings? (a posteriori PT)	Discussion/Conclusion: Revise or revisit or state their programme theory of how the intervention is likely to work’ at the end of the review to explain their findings? (a posteriori PT)	Additional considerations: Indicate whether reported that tool is based on shared consensus across the team or across stakeholders	Additional considerations: Was PT tested?
Backholeret al. [28]			✓	✓						✓	✓		✓
Bambra et al. [29]^a^		✓	✓	✓	✓	✓		✓	✓	✓	✓		✓
Bambra et al. [29]^b^		✓	✓	✓	✓	✓		✓	✓	✓	✓		✓
Beauchamp et al. [33]		✓	✓	✓				✓	✓	✓	✓		✓
Boelsen-Robinson et al. [34]			✓	✓				✓	✓	✓	✓		✓
Brown et al. [36]^c^		✓	✓	✓	✓	✓		✓	✓	✓	✓		
Brown et al. [37]		✓						✓		✓	✓		
Brown et al. [38]			✓							✓	✓		
Brown et al. [39]										✓	✓		
Bull et al. [50]		✓	✓	✓						✓	✓		✓
Ciciriello et al. [58]			✓	✓			✓			✓	✓		✓
Cleland et al. [41]			✓							✓	✓		✓
Ejemot-Nwadiaro et al. [59]			✓	✓		✓							
Everson-Hock et al. [42]			✓	✓				✓	✓	✓	✓		✓
Gardner et al. [43]			✓							✓	✓		✓
Gittelsohn et al. [60]			✓	✓						✓	✓		
Gurol-Urganci et al. [61]			✓	✓									
Hartmann-Boyce et al. [62]		✓	✓	✓					✓	✓	✓		✓
Hill et al. [44]		✓	✓							✓	✓		
Hollands et al. [45]			✓	✓	✓	✓		✓	✓				
Kader et al. [46]										✓	✓		
Kendrick et al. [47]			✓	✓						✓			
Kristjansson et al. [48]			✓	✓		✓		✓	✓	✓	✓		✓
Kroon et al. [63]										✓	✓		
Laba et al. [49]		✓	✓	✓				✓	✓	✓	✓		✓
Laws et al. [50]			✓	✓						✓	✓		✓
Lutge et al. [64]			✓	✓						✓	✓		
Magnee et al. [51]		✓	✓	✓						✓	✓		✓
McGill et al. [52]		✓	✓	✓				✓	✓	✓	✓		✓
McLean et al. [20]	✓	✓	✓	✓	✓	✓		✓	✓	✓	✓	✓	✓
Mizdrak et al. [53]			✓	✓						✓			
Moore et al. [54]		✓	✓	✓				✓	✓	✓	✓		✓
Moredich et al. [55]		✓								✓	✓		
Pega et al. [65]			✓	✓						✓			
Polec et al. [66]	✓	✓	✓	✓	✓	✓		✓	✓	✓	✓	✓	✓
Rojas-Garcia et al. [56]			✓	✓	✓					✓	✓		✓
Sarink et al. [57]			✓	✓	✓	✓		✓	✓	✓	✓		✓
Total	2	15	32	28	8	9	1	15	15	34	31	2	21

Programme theory also includes reference to logic models/frameworks/causal pathway analysis

*PT* programme theory, *SES* socio-economic status

^a^Study 1 review in child population [32, 34]

^b^Study 2 review in adult population [32, 33, 35]

^c^One study reported in two publications [38, 39]

Twenty-one studies (see Table 10) tested their a priori programme theory of how they expected interventions to have different effects on health within or across SES populations *and* revisited or revised their programme theory to explain their review findings.

## Discussion

Interest in the use of programme theory to inform systematic reviews is increasing. In an attempt to enhance the applicability of review findings, reviewers are being encouraged to extend consideration beyond whether an intervention is effective or not, towards examining ‘what interventions work, for whom, and how’ [71].

While the relatively small proportion of systematic reviews (*n* = 108) incorporating considerations of socio-economic inequalities in health is in line with that reported elsewhere [72], it is clear that the push to move systematic reviews away from only considering *if* an intervention works towards a better understanding of *what* works and *how*, is slowly starting to emerge in the literature (see Table 10).

The lack of reference to the equity guidance within systematic reviews incorporating considerations of socio-economic inequalities in health may reflect the short interval between the publication of the guidance and the systematic reviews included in this study. However, given that eight studies in this sample did reference the PRISMA Equity guidance between 2013 and 2016, it may also suggest a lack of awareness of the guidance, or consideration of its relevance and importance, not only among systematic reviewers, but also among journal editors and peer reviewers. Therefore, to deliver better evidence on equity within research syntheses and systematic reviews, not only does the health research community need to increase awareness of equity guidance, but journal editors and peer reviewers also need to be proactive in encouraging reviewers to adopt the equity guidance when undertaking and reporting such reviews [1].

Alternatively, a lack of reference to the equity guidance in the reviews may indicate that reviewers are unsure about how to operationalise the guidance with respect on how the intervention may be expected to work within or across SES populations. Little empirical research has been undertaken on reviewers’ understanding of how to operationalise equity guidance for systematic reviews.

In studies that do use programme theory terminology (e.g., logic model, conceptual framework), the findings here agree with commentators who note that these terms are often inconsistently applied in the literature [16, 18]. No studies explicitly applied the term ‘programme theory’ to describe their assumptions. This supports assertions made elsewhere [16] that the use of theory to inform systematic reviews may not be explicitly articulated.

While it may not necessarily matter whether or not reviewers explicitly label a ‘programme theory’ to describe their understanding of how interventions may or may not work, *not* explicitly labelling it makes it harder to ascertain the extent to which reviewers are either consciously or unconsciously *using* programme theory to inform systematic reviews. The majority of the reviews included in this study described their programme theory in the narrative of the review without explicitly labelling it as a theory or using more graphical representations such as logic models or analytical frameworks. If reviewers are either considering this detail irrelevant to the methods section of their reports, or unconsciously using programme theory, then there is a need for greater clarity on operationalising the use of theory in systematic reviews.

In most of the included studies, programme theory was informed by low-level theory (i.e. assumptions based on supplementary evidence, e.g. empirical or policy). This is consistent with the findings of a previous study on the use of theory in systematic reviews [16]. The lack of reference to more formal theory (e.g. intervention theories such as Whitehead’s [22] typology of policies and interventions, and behaviour change theories such as the Theory of Planned Behavior [73]) to inform reviewers’ assumptions or explanations of whether and how interventions may have differential effects may suggest that reviewers are using programme theory unconsciously. Among the included reviews that used formal theory, the most popular were intervention theories based on the target of the intervention (e.g. individual, community, societal).

While this study set out neither to examine the quality or richness of the programme theory used in systematic reviews nor to establish the fidelity or utility of use of theory, analysing interventions based only on the target of the intervention (i.e. universal versus individual) may not offer sufficient explanation of which *components* of the intervention process may work better for different SES groups and why.

Using only supplementary evidence to *explain* how interventions may work differently across SES populations may weaken the applicability of the review, especially given that it was often unclear as to how the supplementary evidence was identified. This is not limited to systematic reviews with a socio-economic health inequality focus. MacLure [74] observes that there is a strict inclusion and exclusion process set up for the main body of the review, only for other evidence to be brought into play in the discussion in an unsystematic way to interpret the findings.

While Anderson et al. [9] warn that, ‘conclusions drawn about intervention effects based simply on ad hoc criteria, rather than a theoretical understanding of the putative mechanisms of action of the intervention, can sometimes obscure aspects of the intervention that contribute to its effect’, using programme theory in an ad hoc and supplementary way to explain review findings may, therefore, result in conclusions based on poor-quality studies that may have little direct relevance to the topic under review.

The results of this study demonstrate that the use of programme theory to inform socio-economic health inequality considerations in the systematic review process remains in its infancy, is used implicitly, is often fragmented and is not implemented in a systematic way. The PRISMA Equity Extension explanation and elaboration document ([5], ‘Item 3: rationale’) highlights that the explicit reporting of programme theory can guide the reviewer in the choice of methods and synthesis. However, the findings of this study agree with Kneale et al. [12], in suggesting that programme theory is not yet seen as a tool that is integral to the whole review process.

Instead, reviewers are more likely to use programme theory in an ad hoc way at the start (a priori) and end of the review using supplementary evidence rather than to use formal theory or to test their assumptions or explanations of how interventions may or may not work for different SES groups. This may be due to the fact that many reviewers are using programme theory implicitly and therefore are unaware of its potential value in guiding the whole review process.

Using programme theory to inform only an a priori understanding of how reviewers expect the intervention to work across SES populations allows reviewers to ‘tick a box’ in the PRISMA Equity Extension guidance [1]. However, integration of programme theory within the whole review process would provide a more systematic ‘uncovering’ of possible explanations that emerge a posteriori from the review findings for *how* interventions may work differently for different populations.

Establishing review intentions a priori has always been the approach in clinical effectiveness reviews in order to minimise bias [75]. However, a priori assumptions of *how* an intervention may work may not necessarily be supported by the review findings. This may lead to an uninformed interpretation of the problem being imposed at the outset of the review. Furthermore, reviewers may not necessarily identify all the issues relating to *how* an intervention is expected to work a priori, and therefore, a revision of the programme theory may be required [76].

In avoiding the use of programme theory simply becoming what Kneale et al. [12] have described as a ‘tick-box’ exercise in demonstrating compliance with the PRISMA Equity Extension criteria, reviewers need to understand how programme theory can help in moving beyond simply basing their systematic reviews on theory towards securing a theoretical underpinning of the review analysis and synthesis.

The value of a programme theory approach lies in its ability to allow an acceptable, systematic, tested and refined a posteriori *reasoning* rather than post hoc *assumption* of *how* interventions may work. Twenty-one reviews in this study tested their programme theories; in doing so, they present review findings that are *tested* interpretations or explanations, rather than unbiased observations, thus strengthening the validity and applicability of the review findings.

Using programme theory to articulate considerations of *if*, *what* and *how* interventions work for different populations will require reviewers to include more diverse forms of evidence beyond randomised controlled trials. Only 12 reviews in this study included evidence other than quantitative research to support their programme theory, largely using a narrative synthesis approach.

If reviewers of effectiveness studies are to be encouraged to consider health inequalities in systematic reviews, this will require a paradigm shift: they will need to move from a positivist stance in considering only ‘if and what works’ towards a more realist informed way of thinking to consider ‘what works, for whom, and under what circumstance’. It requires a different approach to analysis moving away from a purely observational approach (i.e. how often, how much) to one that incorporates a more interpretive approach. This shift is hugely challenging, requiring skills in understanding theoretical sensitivity and in being able to generate, test and refine possible explanations.

While there is a greater need to understand the advantages and disadvantages of different synthesis approaches, the decision on what synthesis approach to undertake and whether to conduct separate parallel reviews has to be balanced with considerations of time, resources and the skills and expertise within the review team [9]. Given that it is unrealistic to expect all reviewers to accompany each systematic review with a parallel realist review, the challenge is how reviewers of effectiveness studies can incorporate realist principles within reviews of effectiveness to inform considerations of *what* and *how* interventions may work differently across SES populations within a single review.

Establishing an a priori programme theory of *if, what* and *how* an intervention is expected to work across different SES groups, testing and refining it based on the review findings may offer reviewers a way forward. Further research is currently underway by the author (MM) on a theory-led meta-framework of factors associated with differential effects of interventions across SES groups.

### Strengths and limitations of the methodological review

A key strength of this study was the inclusion of full-text searches rather than simply undertaking title and abstract searches alone. Full-text searching took place in order to overcome the limitations associated with the fact that SES analysis may have been undertaken but not reported in journal abstracts. In addition, during the screening phase, if no mention was made in the title or abstract of SES, the full text of the paper was examined.

A further strength was to include studies that were either explicit *or* implicit in their consideration of what and how interventions may have different effects within and across SES populations. This extends a previous study on the use of programme theory [12] and recognises that the articulation of what works, for whom and under what circumstance may not necessarily be recognised as ‘programme theory’ or labelled as a ‘logic model’ or ‘conceptual framework’. Indeed, as this study demonstrates, reviewers rarely identify with these labels even when outlining a programme theory. The involvement of two reviewers to independently extract the data on programme theory aimed to reduce the chances of missing relevant information.

Only systematic reviews published since the introduction of the PRISMA Equity Guidance [1] were included in this study. The short interval between the publication of the guidance and the systematic reviews included in this study may result in a more modest indication of the extent to which reviewers are operationalising the PRISMA guidance in exploring how interventions may result in differential effectiveness within or across SES populations. Studies in which it was not possible to separate out the analysis for SES were excluded; therefore, it may be that other theories relating to what works and how/why they work may have been missed.

Unless explicitly stated in the included studies, this study was unable to assess the way in which different members of the review team or stakeholders contributed to the programme theory. Furthermore, this study only assessed the extent of use of programme theory if reviewers were explicit in reporting their use of it to inform their review processes.

This study did not set out to examine the fidelity, utility or richness of the programme theory. For example, Magnee et al. ([51], e62) described why they assume that differential effectiveness across SES populations may arise, because “more highly educated people may be better equipped to benefit from interventions”, yet they failed to explain in what way or why highly educated people are ‘better equipped’. In another example, Mizdrak et al., ([53], Introduction) stated that they expected differential effectiveness to occur because “low income purchasers may react differently to changes in food price than high income purchasers” but it is not clear in what way or why low income purchasers ‘may react differently’. Therefore, the use of the PRISMA Equity Extension criteria [1] in the quality assessment only allows for an assessment of the quality of the *reporting* of programme theory.

## Conclusions

Given the lack of evidence in primary research on the differential effects of interventions on health across SES groups, Whitehead [22] considers it ‘absolutely imperative’ that a theory-based approach is adopted to guide actions on reducing health inequalities. Despite the PRISMA Equity Extension guidance recommending the use of programme theory, this study demonstrates that use of the guidance to inform considerations of *if*, *what* and *how* interventions lead to differential effects on health within and across SES groups in the systematic review process is not yet widely adopted and is fragmented.

Encouraging reviewers of effectiveness studies to consider health inequalities in systematic reviews requires a paradigm shift in thinking, from a positivist (i.e. ‘if, and what works’), towards a realist informed way of thinking (i.e. ‘what works, for whom, and under what circumstance’). The fact that reviewers are more likely to use programme theory implicitly, in an ad hoc descriptive way, and use supplementary evidence to support their assumptions of how interventions work rather than use more ‘formal’ theories, suggests that reviewers are unconsciously using programme theory and are not fully exploiting its potential in informing the review process.

## Additional file

Additional file 1: Search strategy. (DOCX 15 kb)

## Abbreviations

3ie: International Initiative for Impact Evaluation database
PICO: Population, intervention, comparator and outcome
PRISMA: Preferred Reporting Items for Systematic Reviews and Meta-Analysis
SES: Socio-economic status

## References

[1] Welch V, Petticrew M, Tugwell P, Moher D, O'Neill J, Waters E, White H (2012). PRISMA-Equity 2012 Extension: reporting guidelines for systematic reviews with a focus on health equity. PLoS Med.

[2] Tripney J, Roulstone A, Hogrebe N, Vigurs C, Schmidt E, Stewart R (2015). Interventions to improve the labour market situation of adults with physical and/or sensory disabilities in low- and middle-income countries: a systematic review.

[3] Tugwell P, Petticrew M, Kristjansson E, Welch V, Ueffing E, Waters E, Bonnefoy J, Morgan A, Doohan E, Kelly MP (2010). Assessing equity in systematic reviews: realising the recommendations of the Commission on Social Determinants of Health. BMJ.

[4] Ueffing ETP, Welch V, Petticrew M, Kristjansson E, for the Campbell and Cochrane Equity Methods Group (2012). Equity checklist for systematic review authors.

[5] Welch V, Petticrew M, Petkovic J, Moher D, Waters E, White H, Tugwell P (2016). Extending the PRISMA statement to equity-focused systematic reviews (PRISMA-E 2012): explanation and elaboration. J Clin Epidemiol.

[6] Welch V, Brand K, Kristjansson E, Smylie J, Wells G, Tugwell P (2012). Systematic reviews need to consider applicability to disadvantaged populations: inter-rater agreement for a health equity plausibility algorithm. BMC Med Res Methodol.

[7] MDR M (2015). Health inequalities and evidence synthesis as part of the CLAHRC NWC. In: Position Statement.

[8] Davies KS. Formulating the evidence based practice question: a review of the frameworks. Evid Based Libr Inf Pract. 2011;6(2).

[9] Anderson LM, Oliver S, Michie S, Rehfuss E, Noyes J, Shemilt I (2013). Investigating complexity in systematic reviews of interventions using a spectrum of methods. J Clin Epidemiol.

[10] Davidoff F, Dixon-Woods M, Leviton L, Michie S (2015). Demystifying theory and its use in improvement. BMJ Qual Saf.

[11] Funnells SCRP, J. (2011). Purposeful program theory: effective use of theories of change and logic models.

[12] Kneale D, Thomas J, Harris K (2015). Developing and optimising the use of logic models in systematic reviews: exploring practice and good practice in the use of programme theory in reviews. PLoS One.

[13] Popay J, Sowden A, Petticrew M, Arai L, Rodgers M, Britten N, Roen K, Duffy S (2006). Guidance on the conduct of narrative synthesis in systematic reviews. A product from the ESRC methods programme.

[14] Noyes JL, Lewin S: Supplemental guidance on selecting a method of qualitative evidence synthesis, and integrating qualitative evidence with Cochrane intervention reviews. In: Supplementary guidance for inclusion of qualitative research in Cochrane systematic reviews of interventions version 1 (updated August 2011). edn. Edited by Noyes J, HannesK, Harden A, Harris J, Lewin S, Lockwood C.: Cochrane Collaboration Qualitative Methods Group; 2011.

[15] Tranfield D, Denyer D, Smart P (2003). Towards a methodology for developing evidence-informed management knowledge by means of systematic review. Br J Manag.

[16] Noyes J, Hendry M, Booth A, Chandler J, Lewin S, Glenton C, Garside R (2016). Current use was established and Cochrane guidance on selection of social theories for systematic reviews of complex interventions was developed. J Clin Epidemiol.

[17] Petticrew M (2015). Time to rethink the systematic review catechism? Moving from ‘what works’ to ‘what happens’. Syst Rev.

[18] Maden M (2016). Consideration of health inequalities in systematic reviews: a mapping review of guidance. Syst Rev.

[19] Guise JM, Chang C, Viswanathan M, Glick S, Treadwell J, Umscheid C, Whitlock E, Fu R, Berliner E, Paynter R, Anderson J, Motu’apuaka M, Trikalinos T (2014). Systematic reviews of complex multicomponent health care interventions. Research white paper.

[20] McLean S, Gee M, Booth A, Salway S, Nancarrow S, Cobb M, Bhanbhro S. Targeting the Use of Reminders and Notifications for Uptake by Populations (TURNUP): a systematic review and evidence synthesis (structured abstract). In: Health technology assessment database: Health Services and Delivery Research; 2014. 25642537

[21] Baxter S, Killoran A, Kelly MP, Goyder E (2010). Synthesizing diverse evidence: the use of primary qualitative data analysis methods and logic models in public health reviews. Public Health.

[22] Whitehead M (2007). A typology of actions to tackle social inequalities in health. J Epidemiol Community Health.

[23] Cochrane methodology reviews [methodology.cochrane.org].

[24] Moher D, Liberati A, Tetzlaff J, Altman DG (2009). Preferred Reporting Items for Systematic Reviews and Meta-analyses: the PRISMA statement. BMJ.

[25] Gentles SJ, Charles C, Nicholas DB, Ploeg J, McKibbon KA (2016). Reviewing the research methods literature: principles and strategies illustrated by a systematic overview of sampling in qualitative research. Syst Rev.

[26] Lilford RJ, Richardson A, Stevens A, Fitzpatrick R, Edwards S, Rock F, Hutton JL (2001). Issues in methodological research: perspectives from researchers and commissioners. Health Technol Assess.

[27] Snilstveit B (2012). Systematic reviews: from ‘bare bones’ reviews to policy relevance. J Dev Effect.

[28] Backholer K, Sarink D, Beauchamp A, Keating C, Loh V, Peeters A (2014). The effect of a sugar sweetened beverage tax among different socioeconomic groups: a systematic review. Obes Res Clin Pract.

[29] Bambra C, Hillier F, Cairns-Nagi J, Kasim A, Moore H, Summerbell C. How effective are interventions at reducing socioeconomic inequalities in obesity among children and adults? Two systematic reviews (structured abstract). In: Health technology assessment database: Public Health Research; 2015. 25654155

[30] Hillier-Brown FC, Bambra CL, Cairns JM, Kasim A, Moore HJ, Summerbell CD (2014). A systematic review of the effectiveness of individual, community and societal-level interventions at reducing socio-economic inequalities in obesity among adults. Int J Obes.

[31] Hillier-Brown FC, Bambra CL, Cairns J-M, Kasim A, Moore HJ, Summerbell CD: A systematic review of the effectiveness of individual, community and societal level interventions at reducing socioeconomic inequalities in obesity amongst children. BMC Public Health 2014, 14(1):834-834. 10.1186/1471-2458-14-834 PMC4137097 25113624

[32] Cairns J-M, Bambra C, Hillier-Brown FC, Moore HJ, Summerbell CD (2015). Weighing up the evidence: a systematic review of the effectiveness of workplace interventions to tackle socio-economic inequalities in obesity. J Public Health.

[33] Beauchamp A, Backholer K, Magliano D, Peeters A (2014). The effect of obesity prevention interventions according to socioeconomic position: a systematic review (provisional abstract). Obes Rev.

[34] Boelsen-Robinson T, Peeters A, Beauchamp A, Chung A, Gearon E, Backholer K (2015). A systematic review of the effectiveness of whole-of-community interventions by socioeconomic position. Obes Rev.

[35] Brown TJ, Todd A, O'Malley C, Moore HJ, Husband AK, Bambra C, Kasim A, Sniehotta FF, Steed L, Smith S (2016). Community pharmacy-delivered interventions for public health priorities: a systematic review of interventions for alcohol reduction, smoking cessation and weight management, including meta-analysis for smoking cessation. BMJ Open.

[36] Brown TJ, Todd A, O'Malley CL, Moore HJ, Husband AK, Bambra C, Kasim A, Sniehotta FF, Steed L, Summerbell CD. Community pharmacy interventions for public health priorities: a systematic review of community pharmacy-delivered smoking, alcohol and weight management interventions (structured abstract). In: Health technology assessment database: Public Health Research; 2016. 26962603

[37] Brown T, Platt S, Amos A (2014). Equity impact of population-level interventions and policies to reduce smoking in adults: a systematic review. Drug Alcohol Depend.

[38] Brown T, Platt S, Amos A (2014). Equity impact of European individual-level smoking cessation interventions to reduce smoking in adults: a systematic review. Eur J Pub Health.

[39] Brown T, Platt S, Amos A (2014). Equity impact of interventions and policies to reduce smoking in youth: systematic review. Tob Control.

[40] Bull ER, Dombrowski SU, McCleary N, Johnston M (2014). Are interventions for low-income groups effective in changing healthy eating, physical activity and smoking behaviours? A systematic review and meta-analysis. BMJ Open.

[41] Cleland V, Granados A, Crawford D, Winzenberg T, Ball K (2013). Effectiveness of interventions to promote physical activity among socioeconomically disadvantaged women: a systematic review and meta-analysis. Obes Rev.

[42] Everson-Hock ES, Johnson M, Jones R, Woods HB, Goyder E, Payne N, Chilcott J (2013). Community-based dietary and physical activity interventions in low socioeconomic groups in the UK: a mixed methods systematic review. Prev Med.

[43] Gardner MP, Adams A, Jeffreys M (2013). Interventions to increase the uptake of mammography amongst low income women: a systematic review and meta-analysis. PLoS One [Electronic Resource].

[44] Hill S, Amos A, Clifford D, Platt S (2014). Impact of tobacco control interventions on socioeconomic inequalities in smoking: review of the evidence. Tob Control.

[45] Hollands GJ, Shemilt I, Marteau TM, Jebb SA, Lewis HB, Wei Y, Higgins JP, Ogilvie D. Portion, package or tableware size for changing selection and consumption of food, alcohol and tobacco. Cochrane Database Syst Rev. 2015;(9):Cd011045. 10.1002/14651858.CD011045.pub2 PMC4579823 26368271

[46] Kader M, Sundblom E, Elinder LS (2015). Effectiveness of universal parental support interventions addressing children's dietary habits, physical activity and bodyweight: a systematic review. Prev Med.

[47] Kendrick D, Young B, Mason-Jones AJ, Ilyas N, Achana FA, Cooper NJ, Hubbard SJ, Sutton AJ, Smith S, Wynn P (2013). Home safety education and provision of safety equipment for injury prevention (review). Evid Based Child Health Cochrane Rev J.

[48] Kristjansson E, Francis DK, Liberato S, Benkhalti Jandu M, Welch V, Batal M, Greenhalgh T, Rader T, Noonan E, Shea B *et al*: Food supplementation for improving the physical and psychosocial health of socio-economically disadvantaged children aged three months to five years. In: Cochrane Database Syst Rev: John Wiley & Sons, Ltd; 2015. 10.1002/14651858.CD009924.pub2 PMC6885042 25739460

[49] Laba TL, Bleasel J, Brien JA, Cass A, Howard K, Peiris D, Redfern J, Salam A, Usherwood T, Jan S (2013). Strategies to improve adherence to medications for cardiovascular diseases in socioeconomically disadvantaged populations: a systematic review (provisional abstract). Int J Cardiol.

[50] Laws R, Campbell KJ, van der Pligt P, Russell G, Ball K, Lynch J, Crawford D, Taylor R, Askew D, Denney-Wilson E: The impact of interventions to prevent obesity or improve obesity related behaviours in children (0-5 years) from socioeconomically disadvantaged and/or indigenous families: a systematic review. BMC Public Health 2014, 14(1):779–779 10.1186/1471-2458-14-779 PMC4137086 25084804

[51] Magnee T, Burdorf A, Brug J, Kremers SP, Oenema A, van Assema P, Ezendam NP, van Genugten L, Hendriksen IJ, Hopman-Rock M (2013). Equity-specific effects of 26 Dutch obesity-related lifestyle interventions. Am J Prev Med.

[52] McGill R, Anwar E, Orton L, Bromley H, Lloyd-Williams F, O'Flaherty M, Taylor-Robinson D, Guzman-Castillo M, Gillespie D, Moreira P *et al*: Are interventions to promote healthy eating equally effective for all? Systematic review of socioeconomic inequalities in impact. BMC Public Health 2015, 15(1):457-457. 10.1186/s12889-015-1781-7 PMC4423493 25934496

[53] Mizdrak A, Scarborough P, Waterlander WE, Rayner M (2015). Differential responses to food price changes by personal characteristic: a systematic review of experimental studies. PLoS ONE [Electronic Resource].

[54] Moore GF, Littlecott HJ, Turley R, Waters E, Murphy S (2015). Socioeconomic gradients in the effects of universal school-based health behaviour interventions: a systematic review of intervention studies. BMC Public Health.

[55] Moredich CA, Kessler TA (2014). Physical activity and nutritional weight loss interventions in obese, low-income women: an integrative review (provisional abstract). J Midwifery Womens Health.

[56] Rojas-Garcia A, Ruiz-Perez I, Rodriguez-Barranco M, Goncalves Bradley DC, Pastor-Moreno G, Ricci-Cabello I (2015). Healthcare interventions for depression in low socioeconomic status populations: a systematic review and meta-analysis. Clin Psychol Rev.

[57] Sarink D, Peeters A, Freak-Poli R, Beauchamp A, Woods J, Ball K, Backholer K (2016). The impact of menu energy labelling across socioeconomic groups: a systematic review. Appetite.

[58] Ciciriello S, Johnston Renea V, Osborne Richard H, Wicks I, de Kroo T, Clerehan R, O'Neill C, Buchbinder R. Multimedia educational interventions for consumers about prescribed and over-the-counter medications. In: Cochrane Database Syst Rev: John Wiley & Sons, Ltd; 2013. 10.1002/14651858.CD008416.pub2 PMC11222367 23633355

[59] Ejemot-Nwadiaro Regina I, Ehiri John E, Arikpo D, Meremikwu Martin M, Critchley Julia A. Hand washing promotion for preventing diarrhoea. In: Cochrane database of systematic reviews: John Wiley & Sons, Ltd; 2015. 10.1002/14651858.CD004265.pub3 PMC4563982 26346329

[60] Gittelsohn J, Lee-Kwan SH, Batorsky B (2013). Community-based interventions in prepared-food sources: a systematic review. Prev Chronic Dis.

[61] Gurol-Urganci I, de Jongh T, Vodopivec-Jamsek V, Atun R, Car J. Mobile phone messaging reminders for attendance at healthcare appointments. In: Cochrane Database Syst Rev: John Wiley & Sons, Ltd; 2013. 10.1002/14651858.CD007458.pub3 PMC6485985 24310741

[62] Hartmann-Boyce J, Jebb SA, Fletcher BR, Aveyard P (2015). Self-help for weight loss in overweight and obese adults: systematic review and meta-analysis. Am J Public Health.

[63] Kroon Féline PB, van der Burg Lennart RA, Buchbinder R, Osborne Richard H, Johnston Renea V, Pitt V. Self-management education programmes for osteoarthritis. In: Cochrane Database Syst Rev: John Wiley & Sons, Ltd; 2014. 10.1002/14651858.CD008963.pub2 PMC11104559 24425500

[64] Lutge Elizabeth E, Wiysonge Charles S, Knight Stephen E, Sinclair D, Volmink J. Incentives and enablers to improve adherence in tuberculosis. In: Cochrane Database Syst Rev: John Wiley & Sons, Ltd; 2015. 10.1002/14651858.CD007952.pub3 PMC4563983 26333525

[65] Pega F, Carter K, Blakely T, Lucas Patricia J. In-work tax credits for families and their impact on health status in adults. In: Cochrane Database Syst Rev: John Wiley & Sons, Ltd; 2013. 10.1002/14651858.CD009963.pub2 PMC11649156 23921458

[66] Augustincic Polec L, Petkovic J, Welch VA, Ueffing E, Ghogomu ET, Pardo JP, Grabowsky M, Attaran A, Wells GA, Tugwell P: Strategies to increase the ownership and use of insecticide-treated bednets to prevent malaria. In Cochrane Database Syst Rev: John Wiley & Sons, Ltd; 2015 10.1002/14651858.CD009186.pub2 PMC7025867 25822171

[67] Cutrona SL, Choudhry NK, Stedman M, Servi A, Liberman JN, Brennan T, Fischer MA, Brookhart MA, Shrank WH (2010). Physician effectiveness in interventions to improve cardiovascular medication adherence: a systematic review. J Gen Intern Med.

[68] White M, Adams J, Heywood P, Barbones S (2009). How and why do interventions that increase health overall widen inequalities within populations?. Health, Inequality and Public Health.

[69] Lorenc T, Petticrew M, Welch V, Tugwell P (2013). What types of interventions generate inequalities? Evidence from systematic reviews. J Epidemiol Community Health.

[70] Arblaster L, Lambert M, Entwistle V, Forster M, Fullerton D, Sheldon T, Watt I (1996). A systematic review of the effectiveness of health service interventions aimed at reducing inequalities in health. J Health Serv Res Policy.

[71] Petticrew M, Welch V, Tugwell P (2014). ‘It is surely a great criticism of our profession...’ the next 20 years of equity-focused systematic reviews. J Epidemiol Community Health.

[72] Welch V, Petticrew M, Ueffing E, Benkhalti Jandu M, Brand K, Dhaliwal B, Kristjansson E, Smylie J, Wells GA, Tugwell P (2012). Does consideration and assessment of effects on health equity affect the conclusions of systematic reviews? A methodology study. PLoS One.

[73] Azjen I (1991). The theory of planned behavior. Organ Behav Hum Decis Process.

[74] MacLure M. ‘Clarity bordering on stupidity’: Where’s the quality in systematic review? In: British Educational Research Association annual conference. Manchester; 2004.

[75] Cochrane Handbook for Systematic Reviews of Intervetions.: The Cochrane Collaboration; 2011.

[76] Rohwer A, Booth A, Pfadenhauer L, Brereton L, Gerhardus A, Mozygemba K, Oortwijn W, Tummers M, Van Der Wilt GJ, Rehfuess E (2016). Guidance on the use of logic models in health technology assessments of complex interventions.

[77] Dahlgren G, Whitehead, M.: Tackling inequalities in health: what can we learn from what has been tried? In: Working paper prepared for the Kings Fund International Seminar on Tackling Inequalities in Health*.* Ditchley Park, Oxfordshire; 1993.

[78] Sutton S, Baum A, Johnston M, Sutton S (2004). Determinants of health related behaviours: theoretical and methodological issues. The SAGE handbook of Health Psychology.

