# Supplementary material for: Use of programme theory to understand the differential effects of interventions across socio-economic groups in systematic reviews—a systematic methodology review
Source: Syst Rev. 2017 Dec 29;6:266. doi: 10.1186/s13643-017-0638-9 (PMC5747153; doi:10.1186/s13643-017-0638-9)
Supplement: Additional file 1: — Search strategy. (DOCX 15 kb) [file 13643_2017_638_MOESM1_ESM.docx]

**Additional file 1: Search strategy**

| **Resource** | **Search strategy** |
| --- | --- |
| MEDLINE (Ovid) | 1 exp Meta-Analysis/  2 systematic review*.tw.  3 meta-analys*.tw.  4 ((narrative or integrative or mixed-method) adj2 (review* or synthesis)).tw.  5 ((evidence or knowledge) adj synthes*).tw.  6 (cochrane adj2 review*).tw.  7 1 or 2 or 3 or 4 or 5 or 6  8 exp *Educational Status/  9 exp *Socioeconomic Factors/  10 exp *Health Status Disparities/  11 exp *Income/  12 exp *Employment/  13 exp *Social Class/  14 socioeconomic*.mp.  15 disadvantaged.mp.  16 deprived.mp.  17 "low income".mp.  18 "educational status".mp.  19 "occupational status".mp.  20 ((poverty or income or educational* or occupation* or "low income" or social) adj2 (analysis or disadvantage* or specific or difference* or factor* or inequalit* or depriv* or inequit* or disparit*)).mp.  21 ((occupation* or income* or education* or social) adj3 (grade* or level* or status)).mp.  22 8 or 9 or 10 or 11 or 12 or 13 or 14 or 15 or 16 or 17 or 18 or 19 or 20 or 21  23 7 and 22  24 limit 22 to systematic reviews or meta-analysis  25 23 or 24  26 limit 25 to (english language and yr="2013 - 2016") |
| CINAHL | S1 (MH "Meta Analysis")  S2 (MH "Systematic Review")  S3 TX (“systematic review*” OR meta-analys* OR “Cochrane review” OR "meta-synthesis" OR "integrative review" OR “evidence synthesis” or “narrative review*” OR “knowledge synthesis” OR “mixed-method review*”)  S4 S1 OR S2 OR S3  S5 (MM "Socioeconomic Factors+")  S6 (MM "Educational Status")  S7 (MM "Health Status Disparities")  S8 (MM "Income+")  S9 (MM "Employment Status")  S10 (MM "Social Class+")  S11 TX Socioeconomic OR socio-economic OR disadvantaged OR depriv* OR “low income” OR “educational status” OR “occupational status”  S12 TX (poverty or income or educational* or occupation* or "low income" or social) N2 (analysis or disadvantage* or specific or difference* or factor* or inequalit* or depriv* or inequit* or disparit*)  S13 TX (occupation* or income* or education* or social) N3 (grade* or level* or status)  S14 S5 OR S6 OR S7 OR S8 OR S9 OR S10 OR S11 OR S12 OR S13  S15 (S4 AND S14)  S16 (S4 AND S14) Limiters - Publication Year: 2013-2016 |
| Google Scholar | "socioeconomic status" "systematic review" "differential effect*"  "socioeconomic status" "narrative review" "differential effect*"  "socioeconomic status" "meta-analysis" "differential effect*" |
| Cochrane | #1MeSH descriptor: [Educational Status] explode all tre  #2MeSH descriptor: [Socioeconomic Factors] explode all trees  #3MeSH descriptor: [Health Status Disparities] explode all trees  #4MeSH descriptor: [Income] explode all trees  #5MeSH descriptor: [Employment] explode all trees  #6MeSH descriptor: [Social Class] explode all trees  #7Socioeconomic or socio-economic or disadvantaged or depriv* or "low income" or "educational status" or "occupational status"  #8(poverty or income or educational* or occupation* or "low income" or social) near/2 (analysis or disadvantage* or specific or difference* or factor* or inequalit* or depriv* or inequit* or disparit*)  #9(occupation* or income* or education* or social) near/3 (grade* or level* or status)  #10 #1 or #2 or #3 or #4 or #5 or #6 or #7 or #8 or #9 Publication Year from 2013 to 2016 |
| Campbell | (Socioeconomic OR socio-economic OR disadvantaged OR depriv* OR “low income” OR “educational status” OR “occupational status” OR “occupational level” OR “educational level” OR “income level” OR “social class*” OR “social position*” OR poverty OR “social* disadvantage” OR “social inequalit*” OR “social inequit*” OR “social disparit*”) all text Limit to reviews, 2013-2016 |
| 3ie | Limit to effectiveness review, 2013-2016 |
| Database of promoting health effectiveness reviews (DoPHER) | “Socioeconomic” OR “socio-economic” OR “disadvantaged” OR “depriv*” OR “low income” OR “educational status” OR “occupational status” OR “occupational level” OR “educational level” OR “income level” OR “social class*” OR “social position*” OR poverty OR “social* disadvantage” OR “social inequalit*” OR “social inequit*” OR “social disparit*” |
